# Supplementary material for: Seminoma and Embryonal Carcinoma Footprints Identified by Analysis of Integrated Genome-Wide Epigenetic and Expression Profiles of Germ Cell Cancer Cell Lines
Source: PLoS One. 2014 Jun 2;9(6):e98330. doi: 10.1371/journal.pone.0098330 (PMC4041891; doi:10.1371/journal.pone.0098330)
Supplement: File S1 — ZIP file containing DMRforPairs output for significant regions. Please start from the html files. (ZIP) [file pone.0098330.s008.zip › figures/37081.pdf]

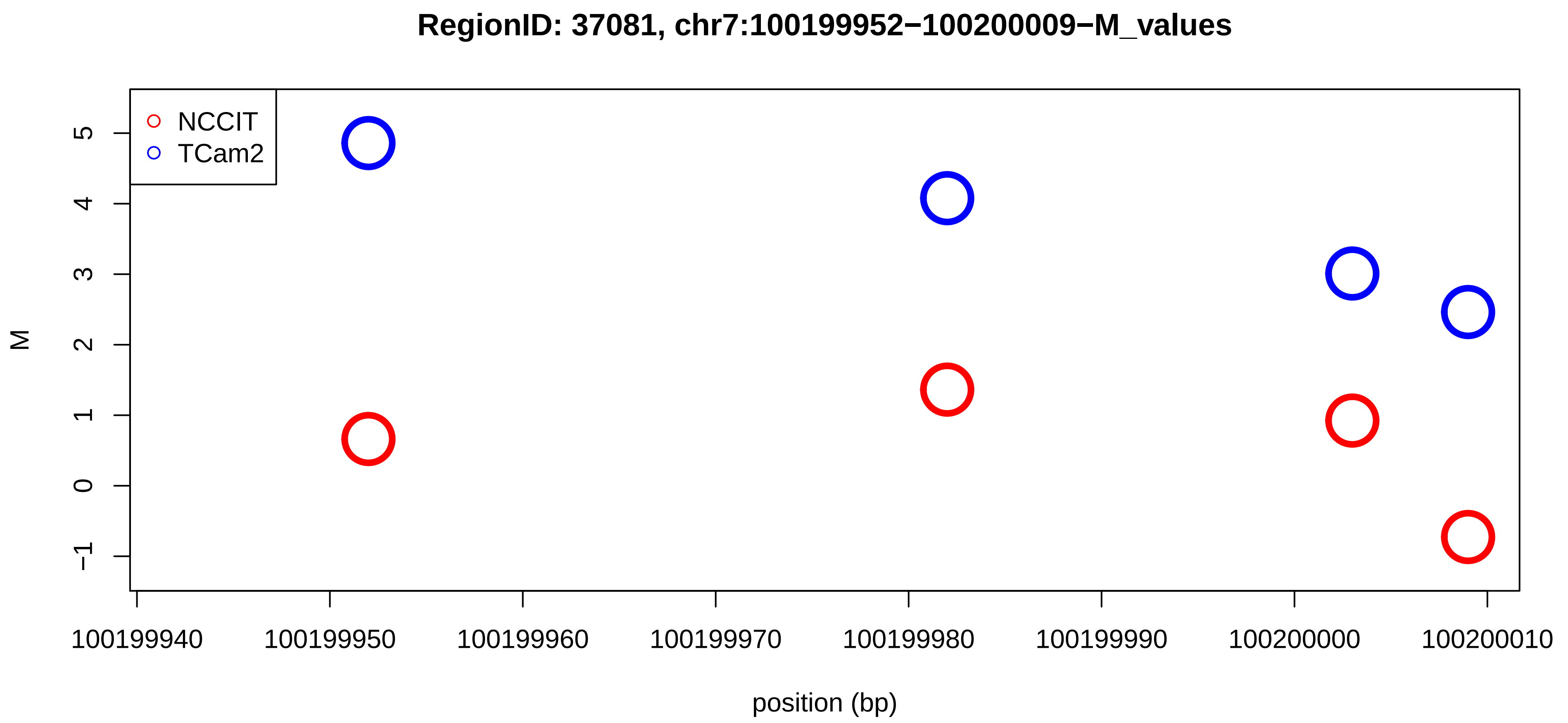

RegionID: 37081, chr7:100199952−100200009−M_values
NCCIT
TCam2
M
5
4
3
2
1
0
−1
100199940
100199950
100199960
100199970
100199980
100199990
100200000
100200010
position (bp)


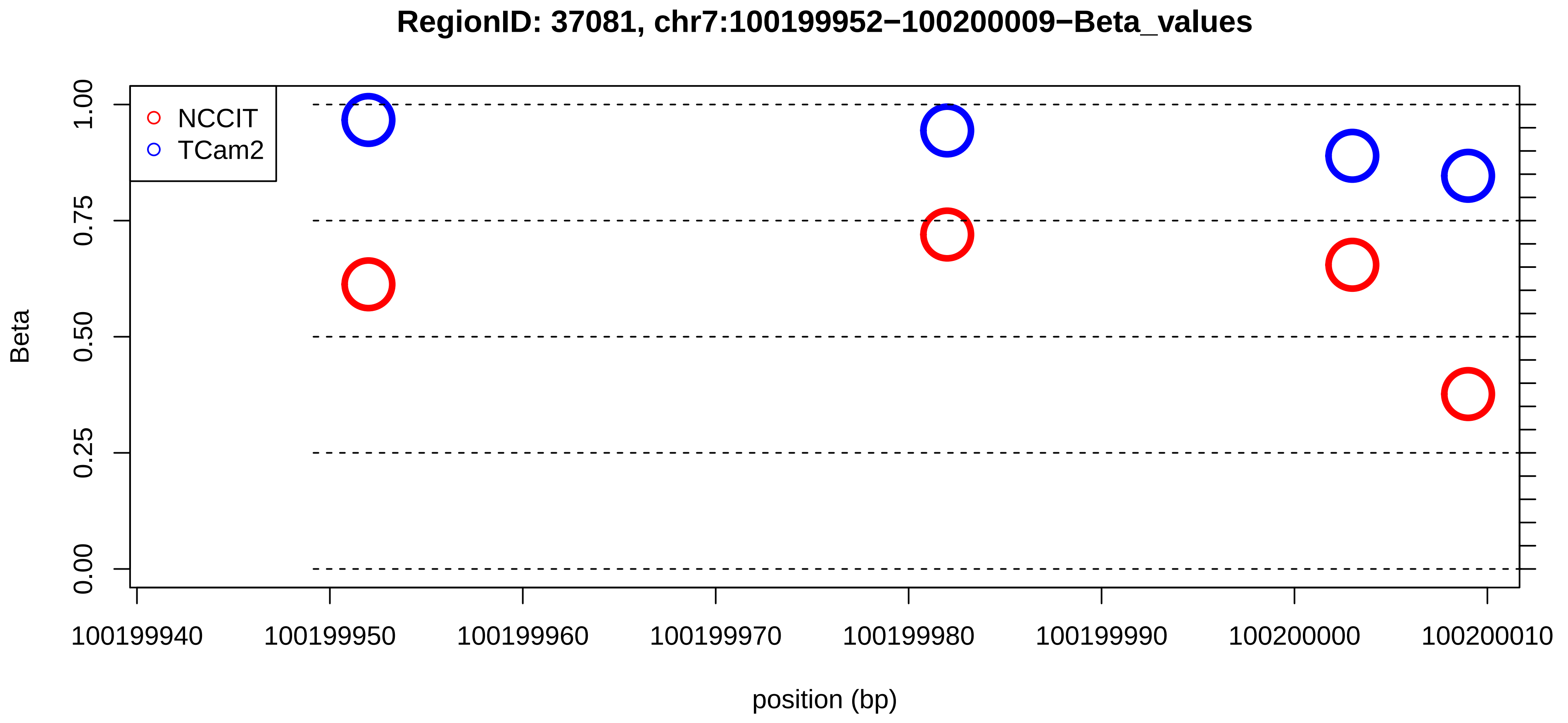

RegionID: 37081, chr7:100199952−100200009−Beta_values
NCCIT
TCam2
Beta
1.00
0.75
0.50
0.25
0.00
100199940
100199950
100199960
100199970
100199980
100199990
100200000
100200010
position (bp)
